# A Retinoid X Receptor Agonist Directed to the Large Intestine Ameliorates T-Cell-Mediated Colitis in Mice

**DOI:** 10.3389/fphar.2021.715752

**Published:** 2021-08-12

**Authors:** Ryohtaroh Matsumoto, Daisuke Takahashi, Masaki Watanabe, Shunsuke Nakatani, Yuta Takamura, Yuji Kurosaki, Hiroki Kakuta, Koji Hase

**Affiliations:** ^1^ Division of Biochemistry, Graduate School of Pharmaceutical Science and Faculty of Pharmacy, Keio University, Tokyo, Japan; ^2^ Division of Pharmaceutical Sciences, Graduate School of Medicine Dentistry and Pharmaceutical Sciences, Okayama University, Okayama, Japan; ^3^ International Research and Development Center for Mucosal Vaccines, The Institute of Medical Science, The University of Tokyo (IMSUT), Tokyo, Japan

**Keywords:** RXR, NEt-3IB, inflammatory bowel disease, colitis, Th1 cells

## Abstract

Retinoid X receptor (RXR) is a nuclear receptor that heterodimerizes with several nuclear receptors, integrating ligand-mediated signals across the heterodimers. Synthetic RXR agonists have been developed to cure certain inflammatory diseases, including inflammatory bowel diseases (IBDs). However, pre-existing RXR agonists, which are lipophilic and readily absorbed in the upper intestine, cause considerable adverse effects such as hepatomegaly, hyperlipidemia, and hypothyroidism. To minimize these adverse effects, we have developed an RXR agonist, NEt-3IB, which has lipophilic and thus poorly absorptive properties. In this study, we evaluated the effects of NEt-3IB in an experimental murine colitis model induced through the adoptive transfer of CD45RB^high^CD4^+^ T cells. Pharmacokinetic studies demonstrated that the major portion of NEt-3IB was successfully delivered to the large intestine after oral administration. Notably, NEt-3IB treatment suppressed the development of T cell-mediated chronic colitis, as indicated by improvement of wasting symptoms, inflammatory infiltration, and mucosal hyperplasia. The protective effect of NEt-3IB was mediated by the suppression of IFN-γ-producing Th1 cell expansion in the colon. In conclusion, NEt-3IB, a large intestine-directed RXR agonist, is a promising drug candidate for IBDs.

## Introduction

Crohn’s disease (CD) is an inflammatory bowel disease (IBD) that is characterized by intermittent abdominal pain, diarrhea, bodyweight loss, and hematochezia (bloody stools) (Ho and Khalil, 2015). Although the exact etiology of CD is unknown, genetic variations and immune dysregulation have been implicated in CD development (De Souza and Fiocchi, 2016). In the gastrointestinal mucosal of patients with CD, inflammation is prolonged by excessive activation of immune cells such as effector T cells and inflammatory macrophages (De Souza and Fiocchi, 2016). For decades, steroids have been used to cure CD, although long-term treatment with these agents causes considerable adverse effects such as edema and sleep disturbance (Carter et al., 2004). More recently, biological agents such as anti-TNF-α antibodies were developed to potently suppress the inflammatory response in CD. However, the cost of biological agents is much higher than that of the classical chemical drugs, and approximately 30% of CD patients fail to respond to therapy using anti-TNF-α antibodies (Adegbola et al., 2018). Moreover, half of the primary responders lose their therapeutic response over time, thereby facing an increased dosage or a revised therapeutic strategy (Adegbola et al., 2018). These facts emphasize the medical need for the development of first-in-class medicines with high efficacy, low cost, and minimal adverse effects.

Retinoid X receptor (RXR) is a nuclear receptor that heterodimerizes with other nuclear receptors such as peroxisome proliferator-activated receptor (PPAR), liver X receptor (LXR), pregnane X receptor (PXR), and NR4A1 (Nur77) (Evans and Mangelsdorf, 2014). Treatment with an RXR agonist can activate these partner nuclear receptors without their own ligand stimulation (Takeuchi et al., 2013; Evans and Mangelsdorf, 2014), a phenomenon termed the “permissive effect.” Activation of these nuclear receptors shows an anti-inflammatory effect, at least partly through mitigating the proliferation and differentiation of inflammatory macrophages and effector T cells (Onuki et al., 2018). In particular, activation of PPARγ by agonist treatment inhibits the differentiation of IFN-γ-producing type 1 helper T (Th1) cells by blocking the JAK–STAT signaling pathway (Natarajan and Bright, 2002; Kanakasabai et al., 2010; Solt et al., 2012). Similarly, activation of LXR inhibits the proliferation of T cells by controlling sterol metabolism via induction of ATP-binding cassette transporter G1 (ABCG1), which is involved in the transmembrane transport of sterols (Bensinger et al., 2008). NR4A1 deficiency is reported to expand the Th1 response because activation of NR4A1 also limits the proliferation of CD4^+^ T cells by controlling oxidative phosphorylation and aerobic glycolysis (Liebmann et al., 2018). Furthermore, treatment with a PXR agonist prevents the expansion of Th1 cells (Dubrac et al., 2010).

**FIGURE 1:**
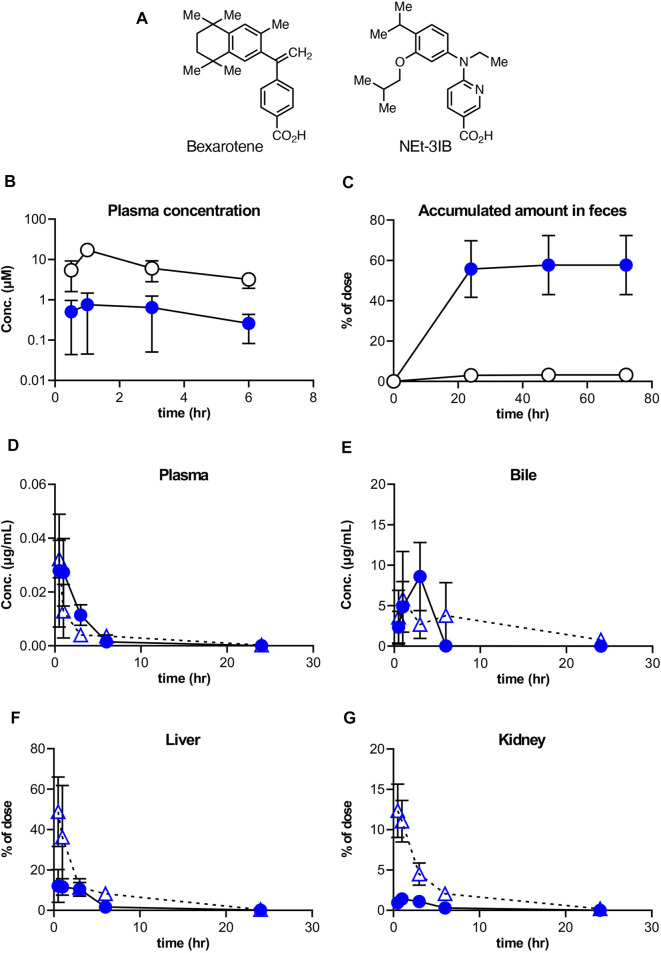
Distribution of NEt-3IB after a single dosage to male mice. **(A, B)** Concentration of bexarotene or NEt-3IB in plasma and feces, respectively. Each compound was orally administered at **(A)** 30 mg/kg and **(B)** 10 mg/kg. **(C–F)** Plasma, bile, liver, and kidney distribution of NEt-3IB after single administration at 1 mg/kg (*i.v*.) and 10 mg/kg (p.o.). Circles and triangles show p.o. and i,v. data, respectively. Data shown are mean [*n* = 5 **(A, C–F)** or 3 **(B)**] ± SD.

RXR agonists can activate these partner receptors simultaneously, and therefore RXR was considered a promising molecular target for inflammatory disorders. Nonetheless, pre-existing RXR agonists have been reported to induce various adverse effects, such as excess bodyweight gain, hepatomegaly, hypothyroidism, and hypertriglyceridemia (Standeven et al., 1997; Liu et al., 2002; de Vries-Van der Weij et al., 2009). To address this issue, we recently developed NEt-3IB, an RXR full agonist with lower lipophilicity compared with pre-existing RXR agonists such as bexarotene (Figure 1A) (Kobayashi et al., 2015). We demonstrated that NEt-3IB induces a low blood concentration after oral administration. Furthermore, using PET/CT imaging, we also observed that NEt-3IB exhibited enterohepatic circulation upon intestinal absorption and was delivered to the lower digestive tract (Kobayashi et al., 2015). These pharmacokinetic properties of NEt-3IB may be beneficial in avoiding systemic adverse effects and delivering the drug to the lower intestine (Kakuta et al., 2012). In this study, we evaluated the anti-inflammatory effects of NEt-3IB in an experimental CD model induced by adoptive transfer of CD45RB^high^CD4^+^ T cells (Powrie et al., 1993). We report that NEt-3IB was persistent in the intestinal lumen after oral administration and ameliorated the wasting and colitis. This effect was attributed to the reduced accumulation of Th1 cells in the colon.

## Materials and Methods

### Chemical Reagents

Bexarotene and NEt-3IB were synthesized as described previously (Boehm et al., 1994; Takamatsu et al., 2008).

### Animals

ICR mice were purchased from Charles River Laboratories Japan (Yokohama, Japan) and maintained under specific pathogen-free (SPF) conditions at Okayama University, Okayama, Japan. C.B-17-*scid*/*scid* and C.B-17^+/+^ mice were purchased from CLEA Japan (Tokyo, Japan) and maintained under SPF conditions at Keio University, Tokyo, Japan. Rag1^*−/−*^ mice were bred and maintained under SPF conditions at Keio University. C.B-17-scid/scid and Rag1^*−/−*^ mice were fed with CE-2 diet (CLEA Japan) before T cell transfer and with MF diet (Oriental Yeast, Tokyo, Japan) containing 0.015% NEt-3IB or regular MF after transfer. Foxp3-hCD2 knock-in mice were bred and maintained under SPF conditions at Keio University. C57BL6/J mice were purchased from CLEA Japan. Foxp3-hCD2 knock-in mice and C57BL6/J mice were fed with AIN-93G diet (Research Diet, New Brunswick, NJ, United States) until their use in experiments at 6–12 weeks old. All animal experiments except for pharmacokinetic studies were approved by the Animal Studies Committees of Keio University.

### Pharmacokinetic Studies

This experiment was conducted in accordance with the Guidelines for Animal Experiments at Department of Animal Resources, Advanced Science Research Center, Okayama University, and all procedures were approved by the Animal Care and Use Committee, Okayama University. The test compounds were administered to 6-week-old ICR male mice (*n* = 5 per group). In the oral administration study, the test compounds (10 mg/10 ml/kg) were suspended in 1% ethanol and 0.5% carboxymethyl cellulose (CMC) in distilled water and administered by oral gavage. In the intravenous administration study, the test compounds (1 mg/5 ml/kg) were dissolved in saline containing 20% WellSolve (Celeste Corporation, Tokyo, Japan) and administered via the tail vein. Blood samples (approximately 0.6–1.0 ml each) were taken from the inferior vena cava under isoflurane anesthesia at 0.5, 1, 3, 6, and 24 h after administration. Then the animals were killed by cervical dislocation. Brain, liver, kidney, bile, and large-intestinal contents were immediately extirpated. Blood treated with heparin in a centrifuge tube was centrifuged at 1,900 × *g* at room temperature for 5 min. To 100 μl of the resulting plasma, 100 μl ice-cold 5.0 mM ammonium acetate solution (adjusted with acetic acid to pH 5.0) and 1.0 ml ice-cold EtOAc were added. The resulting mixture was vortexed for 30 s, kept at room temperature for 10 min, and centrifuged at 1,900 ×*g* at room temperature for 30 s. An 800 μl aliquot of the EtOAc phase was removed and concentrated to dryness in a centrifugal evaporator. The resulting residue was added to 100 μl acetonitrile. Brain, liver, and kidney samples were homogenized in ice-cold EtOAc (500 µL/100 mg tissue) using a Cell Destroyer PS1000 (BMS, Tokyo, Japan) and steel beads (for 5 mm) at 4,260 rpm for 60 s. Bile was collected by washing out the gallbladder with 500 µl EtOAc. The extract was vortexed for 30 s, kept under ultrasonic irradiation at room temperature for 5 min, and centrifuged at 12,000 ×*g* at 4°C for 10 min. A 200 μl aliquot of the EtOAc phase was collected and concentrated to dryness in a centrifugal evaporator. The resulting residue was dissolved by adding 100 μl acetonitrile. Each solution was directly subjected to LC-MS/MS analysis, and the concentration of each compound was determined from the peak area of the sample with reference to a calibration plot obtained with the authentic compound. All contents in the large intestine were collected, and MeOH (1 ml) was added under ultrasonic irradiation at room temperature for 20 min. The resultant suspension was centrifuged at 1,500 ×*g* at 4°C for 20 min. An 800 μl aliquot of the MeOH phase was diluted 10-fold with methanol. Each solution was directly subjected to High Performance Liquid Chromatography (HPLC) analysis, and the concentration of NEt-3IB was determined from the peak area of the sample with reference to a calibration plot obtained with the authentic compound.

### Liquid Chromatography With Tandem Mass Spectrometry 

The API4000 LC-MS/MS system (Applied Biosystems, Toronto, Canada) was used, consisting of an LC-20AD pump, an SPD-20AV UV-Vis spectrophotometric detector, and a CTO-20AC column oven (SHIMADZU, Kyoto, Japan) operating at 40°C. A TSKgel ODS-100V column (2.0 i.d. × 50 mm, 3 μm; TOSOH, Tokyo, Japan) was used. The mobile phase was acetonitrile/H_2_O = 65/35 containing 0.1% formic acid, v/v, and the flow rate was set at 0.2 ml/min.

### High-Performance Liquid Chromatography 

A Shimadzu liquid chromatographic system (Kyoto, Japan) consisting of an LC-10AD pump, an SPD-10AV UV–vis spectrophotometric detector, a CTO-10AS column oven, and a C-R5A Chromatopac was used. An Inertsil ODS-3 column (4.6 mm i.d. × 100 mm, 5 μm, GL Sciences, Tokyo, Japan) with a guard column of Inertsil ODS-3 (4.6 mm i.d. × 10 mm, 5 μm, GL Sciences) was operated at 40°C. The mobile phase was methanol/H_2_O = 85/15 containing 0.1% formic acid, v/v. The flow rate was set at 0.7 ml/min and the absorbance at 260 nm was monitored.

### Induction of Colitis by Adoptive Transfer of CD4^+^ CD45RB^high^ T Cells

Colitis was induced in C.B-17-scid/scid mice by adoptive transfer of CD45RB^high^ T cells. Mojosort Mouse CD 4 T Cell Isolation Kit (BioLegend, San Diego, CA, United States) and biotin-conjugated anti-mouse TER-119 (TER-119; BioLegend) were used to negatively select CD4^+^ T cells of splenocytes from C.B-17^+/+^ mice. Negatively selected CD4^+^ T cells were labeled with Alexa Fluor 647-conjugated anti-mouse CD4 (RM-4-5; BioLegend), BV510-conjugated anti-mouse CD45 (30-F11; BioLegend), and BV421-conjugated anti-mouse CD45RB (16A; BD Horizon, San Jose, CA, United States). Dead cells were labeled with 7-aminoactinomycin D (7-AAD) (BioLegend). CD45^+^CD4^+^CD45RB^high^ live T cells were isolated by FACS Aria III (BD Biosciences San Jose, CA, United States). The C.B-17-scid/scid recipients were each injected with 2 × 10^5^ cells via the tail vein and transferred to conventional conditions in the animal facilities at Keio University. Mice were fed with 0.015% NEt-3IB-containing MF diet or control MF diet for 7 weeks. The body weights of the mice were measured every week, and the fecal diarrhea score was also measured from week 4. The diarrhea score were assessed as follows: normal stool (0), slightly soft (1), soft but formed (2), not formed (3), and liquid stool (4) (Kjellev et al., 2006). The mice were humanely killed at week 7.

### Cell Preparation

Colonic lamina propria (LP) lymphocytes were obtained as follows. Colon tissues were treated with 1 mM dithiothreitol and 20 mM EDTA containing Hank’s Balanced Salt Solutions (all from Nacalai-Tesque, Kyoto, Japan) for 20 min in a shaking incubator (37°C, 200 rpm) to remove epithelial cells. After the tissues were treated once more with 20 mM EDTA containing Hank’s Balanced Salt Solution for 20 min, the tissues were then minced and dissociated with Liberase solution containing 26 units/ml Liberase TM (Roche, Basel, Switzerland), 5 mg/ml DNase I (Sigma Aldrich, St Louis, MO), 2% newborn calf serum (Thermo Fisher Scientific, Waltham, MA, United States), 100 μg/ml penicillin and streptomycin, and 20 mM HEPES in RPMI 1640 medium (all from Nacalai Tesque) at 37°C for 30 min. The cell suspensions were then filtered to obtain single-cell suspensions. Single-cell suspensions from mesenteric lymph nodes (MLNs) was prepared by mechanically crushing MLNs through 100 μm cell strainers (Greiner Bio-One) in RPMI 1640 medium containing 2% newborn calf serum (Thermo Fisher Scientific).

For cell counts, cells were mixed with Precision Count Beads (BioLegend), anti-mouse CD16/32 (93; BioLegend), eFluor 450-conjugated anti-mouse CD45 (30-F11; Thermo Fisher Scientific), and 7-aminoactinomycin D (7-AAD) (BioLegend) and were analyzed by FACS Celesta and FlowJo software version 10 (BD Biosciences).

### Histology

Colonic tissue samples were fixed in Mildform (10% formalin; Wako Pure Chemical Industries, Osaka, Japan) overnight. After fixation, the samples were embedded in paraffin using a Leica EG1160. Samples were cut into 5 µm sections, deparaffinized and rehydrated, and then stained with hematoxylin (Agilent Technologies, Inc., Santa Clara, CA, United States) and eosin (Wako Pure Chemical Industries, Osaka, Japan). Histological colitis scores were reviewed by DT, who was blinded to each experiment. The sections were scored for the presence of crypt abscesses (0–1), the degree of thickness (0–3), and the degree of inflammatory infiltrate (0–3). The maximum score of the histological index was 7.

### Flow Cytometry

For inflammatory T cell analysis of the colitis model, the following monoclonal antibodies were used: BV510-conjugated anti-mouse CD45 (30-F11; BioLegend), redFluor 710-conjugated anti-mouse CD4 (RM4-5; Tonbo Bioscience), BV605-conjugated anti-mouse TCRβ (H57-597; BD Biosciences), BV650-conjugated anti-mouse IFN-γ (XMG1.2; BD Horizon), and BV786-conjugated anti-mouse IL-17A (TC11-18H10.1; BD Horizon). For Treg cell analysis of the colitis model, the following monoclonal antibodies were used: BV510-conjugated anti-mouse CD45 (30-F11; BioLegend), redFluor-710-conjugated anti-mouse CD4 (RM4-5; Tonbo Biosciences, San Diego, CA, United States), FITC-conjugated anti-mouse TCRβ (H57-597; BD Biosciences), and eFluor 660-conjugated anti-mouse Foxp3 (FJK-16s; ThermoFisher Scientific). For macrophage analysis of the colitis model, the following monoclonal antibodies were used: BV510-conjugated anti-mouse CD45 (30-F11; BioLegend), Alexa Fluor 594-conjugated anti-mouse CD3 (17A2; BioLegend), redFluor 710-conjugated anti-mouse B220 (RA3-6B2; Tonbo Biosciences), APC-conjugated anti-mouse Ly-6C (AL-21; BD Pharmagen, San Jose, CA, United States), Alexa Fluor 488-conjugated anti-mouse Ly-6G/Ly-6C (Gr-1; BioLegend), BUV737-conjugated anti-mouse CD11b (M1/70; BD Horizon), BUV 395-conjugated anti-mouse CD11c (HL3; BD Horizon), and PerCP-Cy5.5-conjugated anti-mouse I-A/I-E (M5/114.15.2; BioLegend). For *in vitro* T cell analysis, the following monoclonal antibodies were used: BV510-conjugated anti-mouse CD45 (30-F11; BioLegend), redFluor 710-conjugated anti-mouse CD4 (RM4-5; Tonbo Biosciences), BUV737-conjugated anti-mouse TCRβ (H57-597; BD Horizon), PerCP-Cy5.5-conjugated anti-mouse IFN-γ (XMG1.2; BioLegend), PE-conjugated anti-mouse/human T-bet (O4-46; BD Pharmagen), and eFluor 660-conjugated anti-mouse Foxp3 (FJK-16s; Thermo Fisher Scientific). Dead cells were labeled with Fixable Viability Stain 780 (BD Biosciences). For intracellular cytokine and transcription factor staining, LP lymphocytes and MLN lymphocytes were cultured for 4 h in complete medium (RPMI containing 10% MP Biomedicals, Santa Ana, CA, United States: fetal bovine serum (FBS), Nacalai-Tesque 100 μg/ml penicillin and streptomycin, Thermo Fisher Scientific 55 µM mercaptoethanol, and Nacalai-Tesque 20 mM HEPES), and cultured T cells were cultured for 4 h in half-complete medium (RPMI containing 5% FBS, 100 μg/ml penicillin and streptomycin, 55 µM mercaptoethanol, and 20 mM HEPES), each supplemented with Cell Activation Cocktail (BioLegend) and Protein Transport Inhibitor Cocktail (Thermo Fisher Scientific). Lymphocytes and T cells were first blocked with anti-CD16/32 and then stained with the monoclonal antibodies described above. IC fixation buffer and permeabilization buffer (Thermo Fisher Scientific) were used for intracellular cytokine staining, and the Transcription Factor Buffer Set (BD Pharmagen) was used for transcription factor staining. For absolute cell counts, cells were mixed with Precision Count Beads (BioLegend) and anti-mouse CD16/32 (93; BioLegend) and stained with eFluor 450-conjugated anti-mouse CD45 (30-F11; Thermo Fisher Scientific) and 7-AAD. The stained samples were analyzed using an LSRII or a FACS Celesta (BD Biosciences), and FlowJo software version 10.

### *In vitro* Cultures

CD4^+^ T cells were enriched from splenocytes from C57BL6/J mice or *Foxp3-hCD2* knock-in mice by a negative selection method with the BD iMag cell separation system, using biotin-conjugated antibodies against mouse CD8α (53-6.7; Tonbo Biosciences), CD11b (M1/70; BioLegend), CD11c (N418; Thermo Fisher Scientific), B220 (RA3-6B2; BioLegend), Ly-6C/Ly6-G (Gr-1; BioLegend), TER-119 (TER-119; BioLegend), and Streptavidin Particle Plus (BD IMag). Negatively selected CD4^+^ T cells were labeled with BV605-conjugated anti-mouse CD4 (RM4-5; BioLegend), BV510-conjugated anti-mouse CD44 (IM4; BD Horizon), redFluor 710-conjugated anti-mouse CD45 (30-F11; Tonbo Biosciences), PE-Cy7-conjugated anti-CD25 (PC61.5; Thermo Fisher Scientific), APC-eFluor 780-conjugated anti-mouse NK 1.1 (PK136; Thermo Fisher Scientific), eFluor450-conjugated anti-CD62L (MEL-14; Thermo Fisher Scientific), and APC-conjugated anti-human CD2 (RPA-2.10; BioLegend). Dead cells were labeled with 7-AAD (BioLegend). CD45^+^CD4^+^CD62L^+^CD44^−^NK 1.1^−^Foxp3^−^(hCD2^−^) live naive T cells were isolated by FACSAria III (BD Biosciences). In the experiments described in Figure 4, 1×10^5^ of CD4^+^ naive T cells were cultured in advanced RPMI containing 5% FBS in the presence or absence of NEt-3IB for 3–5 days. For the experiment using CellTrace Violet (Thermo Fisher Scientific), T cells were incubated at 37°C for 20 min with CellTrace Violet before culture. T cells were polarized to the Th1 subtype by the addition of 10 μg/ml anti-mouse IL-4 (Bio X Cell, Lebanon, NH, United States) and 20 ng/ml recombinant mouse IL-2 and IL-12 (BioLegend). The stained samples were analyzed using FACS LSR II (BD Biosciences) and Flowjo software version 10.

### Statistical Analysis

Statistical significance between two groups was calculated by Student’s t-test or Welch’s t-test. In the *in vitro* study, statistical significance between the control group and other groups was calculated by Dunnet’s multiple comparison test or Dunn’s multiple comparison test. *p* < 0.05 indicated statistical significance. Data were output by Prism version 9 (GraphPad, San Diego, CA, United States).

## Results

### NEt-3IB has Low Systemic Transferability and Reaches the Colon

To better understand the pharmacokinetics of NEt-3IB and bexarotene (Figure 1A), another RXR agonist clinically used for the treatment of cutaneous T cell lymphoma (Budgin et al., 2005), we initially measured their concentrations in plasma and feces after a single oral administration. The plasma concentrations of NEt-3IB were much lower than those of bexarotene at any timepoint (0.5–6 h post-administration) (Figure 1B). Correspondingly, the delivery of NEt-3IB to the feces accumulated to approximately 60% of the dosage, whereas that of bexarotene was less than 10% at 24 h post-administration (Figure 1C). These data confirm that orally administered NEt-3IB reaches the colon more efficiently compared with bexarotene due to its low systemic transferability.

To further examine the systemic pharmacokinetics of NEt-3IB, mice received intravenous (i.v.) injection (1 mg/kg) and oral administration (p.o*.*) (10 mg/kg) of NEt-3IB. The concentrations of NEt-3IB in plasma began to decline by 1 h after i.v. or 6 h after p.o. administration, and reciprocally, the concentrations in the bile increased at these time points (Figures 1D,E). On the basis of these observations, we assumed that the liver may excrete NEt-3IB to the intestinal tract via the bile duct. To test this assumption, we measured the concentration of NEt-3IB in the liver and the kidney. Although the concentration of NEt-3IB in the liver promptly decreased, that in the bile gradually increased after i.v. injection (Figure 1F). The hepatic concentration of NEt-3IB peaked immediately after p.o. administration and then gradually declined (Figure 1F). Conversely, NEt-3IB concentration in the kidney rapidly decreased after i.v. administration and remained consistently low after p.o. administration. Thus, NEt-3IB was excreted to the bile in the liver rather than the kidney (Figure 1G).

### NEt-3IB Treatment Alleviates T Cell-dependent Colitis

**FIGURE 2:**
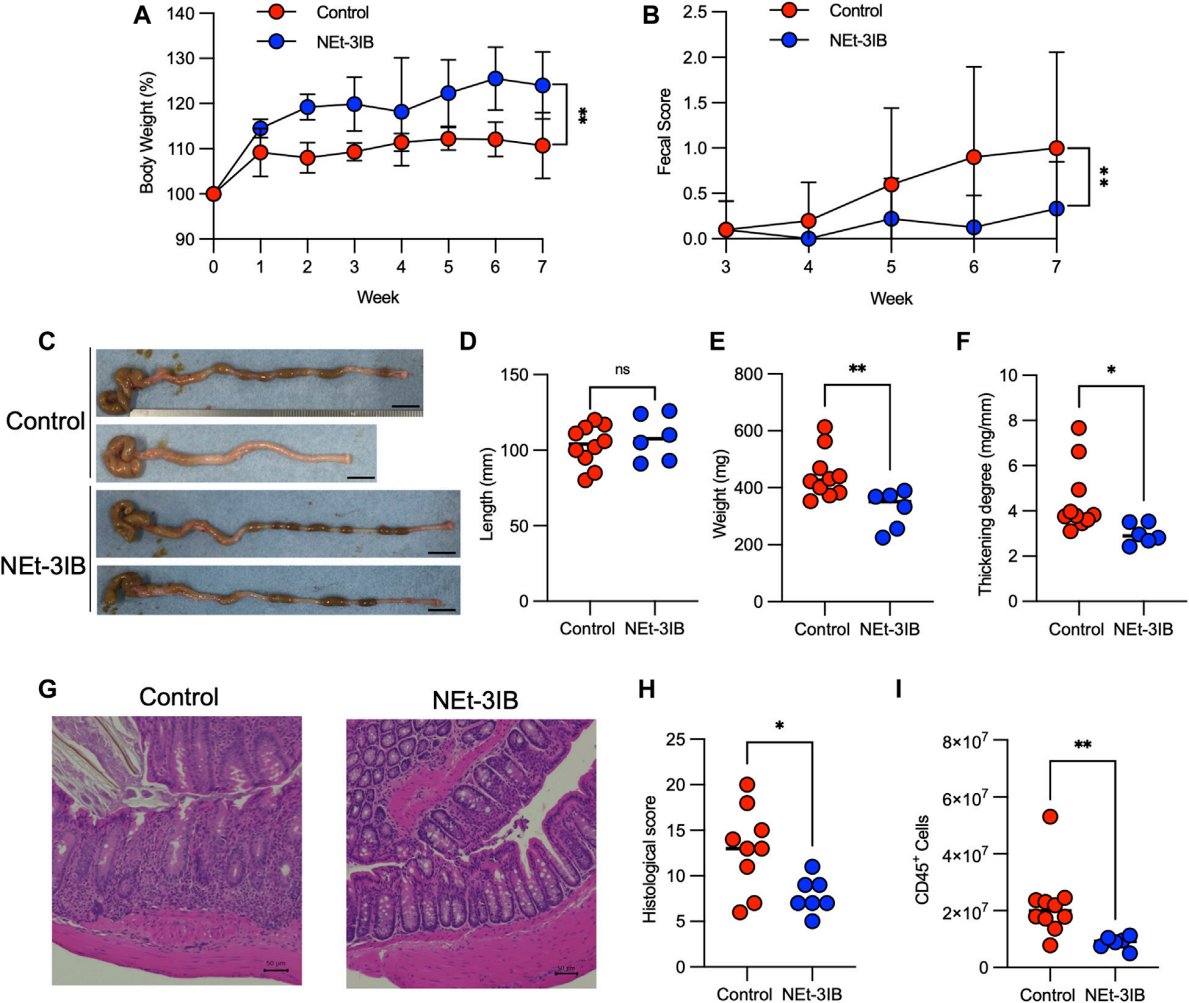
NEt-3IB ameliorates T-cell-dependent experimental colitis. **(A–H)** Experimental colitis was induced by adoptive transfer of CD4^+^CD45RB^high^ T cells into C.B-17 scid/scid mice fed with control or NEt-3IB-containing diet for 7 weeks, and bodyweight loss **(A)** and diarrhea score **(B)** were observed. Data shown are the average. Area under the curve of bodyweight change and fecal score. **(C–F)** Colon length, colon weight, and colon thickening were observed and measured in week 7. Scale bar: 1 cm. **(G)** Colonic specimens were stained with hematoxylin and eosin (HE). **(H)** Histological scores of colonic specimens. **(I)** Total CD45^+^ cells in the colon lamina propria of the CD4^+^ CD45RB^high^ T cell transfer model. *p < 0.05, **p < 0.01.

We subsequently examined the anti-inflammatory effects of NEt-3IB in an experimental colitis model induced by transferring CD4^+^CD45RB^high^ T cells into C.B-17-scid/scid mice and treating with chow containing 0.015% NEt-3IB (approximately 30 mg/kg/day) or with regular chow as a control. In the control group, bodyweight gain was suppressed from 1-week post-transfer onwards (Figure 2A). Meanwhile, the NEt-3IB-treated group gained weight gradually over the course of the experiment, suggesting that NEt-3IB alleviated wasting disease. Similarly, NEt-3IB administration ameliorated diarrhea associated with colitis, even though both groups exhibited only mild symptoms (Figure 2B). Likewise, the colon weight and degree of thickening (colon weight/length) were significantly lower in the NEt-3IB group than that in the control group at 7 weeks after cell transfer (Figures 2C–F). Histopathological examination also showed massive infiltration of mononuclear cells in the colon of the control group but not in the NEt-3IB group (Figure 2G). Furthermore, the control group exhibited more severe crypt loss and edema (Figures 2G,H). Flow cytometry confirmed the massive infiltration of leukocytes in the colon lamina propria (cLP) in the control group, whereas fewer inflammatory infiltrates were observed in the NEt-3IB group (Figure 2I).

To further confirm the pharmacological effects of NEt-3IB, we also evaluated the progressive bodyweight loss and diarrhea in a similar colitis model using *Rag1* ^−/−^ mice as a recipient. The control *Rag1* ^−/−^ recipients exhibited progressive body weight loss in association with severe diarrhea. However, the administration of NEt-3IB significantly mitigated the wasting disease (Supplementary Figure S1A). Furthermore, NEt-3IB improved colitis symptoms, namely, diarrhea, colon weight, colonic wall thickening, crypt loss, and edema (Supplementary Figures S1B–G). Collectively, these data revealed an anti-inflammatory effect of NEt-3IB in the T cell-dependent mouse colitis model.

### NEt-3IB Suppressed the Accumulation of Th1 Cells in the cLP

**FIGURE 3:**
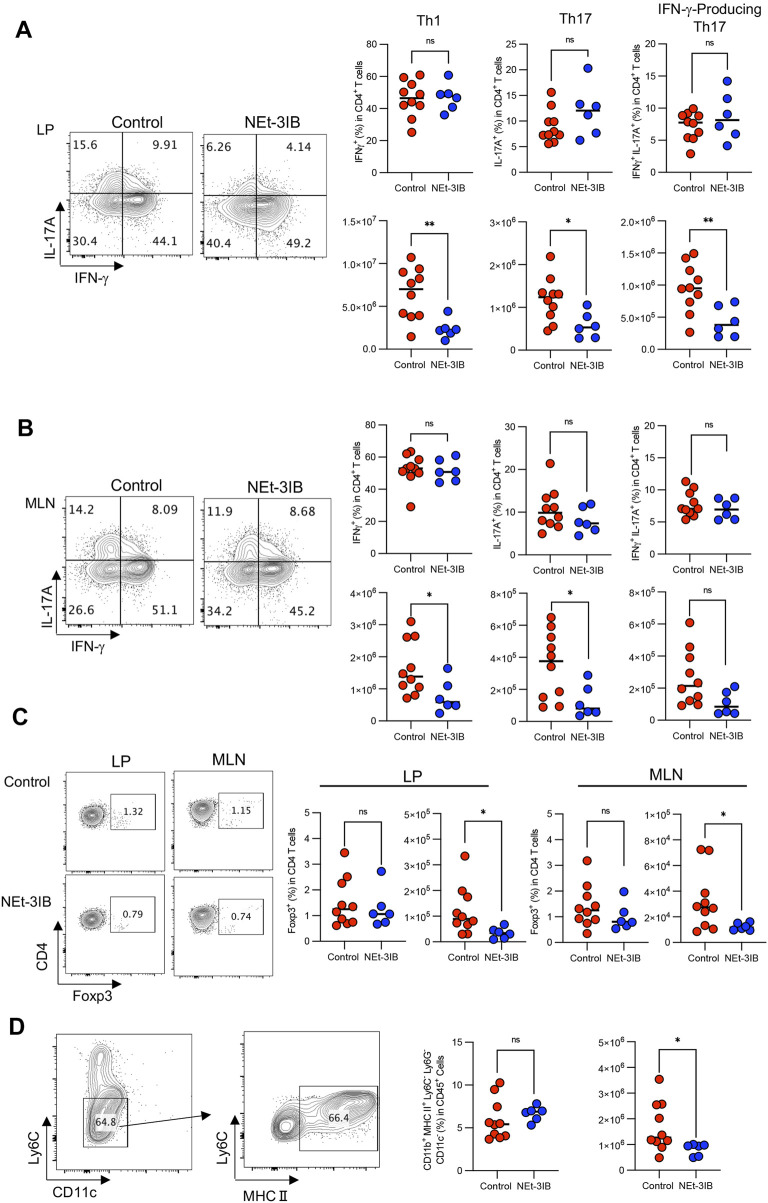
NEt-3IB inhibits inflammatory T cells. **(A)** Frequency and cell number of IFN-γ-producing cells and IL-17A-producing cells in the colon lamina propria. **(B)** Frequency and cell number of Th1 transcription factor T-bet and Th17 transcription factor RORγt-presenting cells of the colon lamina propria and mesenteric lymph nodes. **(C)** Frequency and cell number of Foxp3^+^ regulatory T cells in the colon lamina propria and mesenteric lymph node of T cell-transferred SCID mice. **(D)** Macrophages were gated as CD11b^+^Ly6G^−^Ly6C^−^CD11c^−^MHC2^+^. The right-hand graphs show the frequency of macrophages in CD45^+^ cells and the number of macrophages. **(A–D)** are from CD45RB^high^ T cell transfer model mice at 7 weeks post-transfer.

In the CD4^+^CD45RB^high^ T-cell-transferred colitis model, IFN-γ-producing effector T (T_eff_) cells including conventional T helper 1 (Th1) cells and IFN-γ/IL-17-co-expressing T helper 17 (IFN-γ-producing Th17) cells are responsible for the pathogenesis (Powrie et al., 1993; Sujino et al., 2011). To determine whether NEt-3IB administration affects the development of these T_eff_ cell subsets, we analyzed T cell profiles in the cLP and MLN of the colitis model at 7 weeks post-transfer by flow cytometry after intracellular cytokine staining. In the cLP, the numbers of Th1 cells, Th17 cells, and IFN-γ-producing Th17 cells in the NEt-3IB group were less than half that in the control group, although the frequencies of these cells were comparable between the two groups (Supplementary Figure S2A and Figure 3A). Moreover, Th1 and Th17 cells in the MLN were significantly decreased in the NEt-3IB group compared with the control group, and IFN-γ-producing Th17 cells also tended to be reduced. Of note, we also observed that the number of Foxp3^+^ Treg cells was slightly but significantly decreased in both the cLP and MLN of the NEt-3IB group (Supplementary Figure S2B and Figure 3C). Colonic T_eff_ cell-derived IFN-γ activates macrophages to produce pro-inflammatory cytokines that cause inflammation in the colon (Stout and Bottomly, 1989). Considering that NEt-3IB treatment reduced Th1 cells in the cLP in the colitis model, we analyzed the colonic macrophages at 7 weeks post-transfer. We observed that the total number of macrophages (CD11b^+^ Ly6G^−^Ly6C^−^CD11c^−^ MHC class II^+^) was decreased significantly in the NEt-3IB-treated group compared with the control group (Supplementary Figure S2C and Figure 3D). These data illustrated that NEt-3IB diminishes colitis by inhibiting the accumulation of IFN-γ-producing T_eff_ cells and inflammatory macrophages in the cLP and MLN.

### NEt-3IB Inhibits the Differentiation and Proliferation of Th1 Cells

**FIGURE 4:**
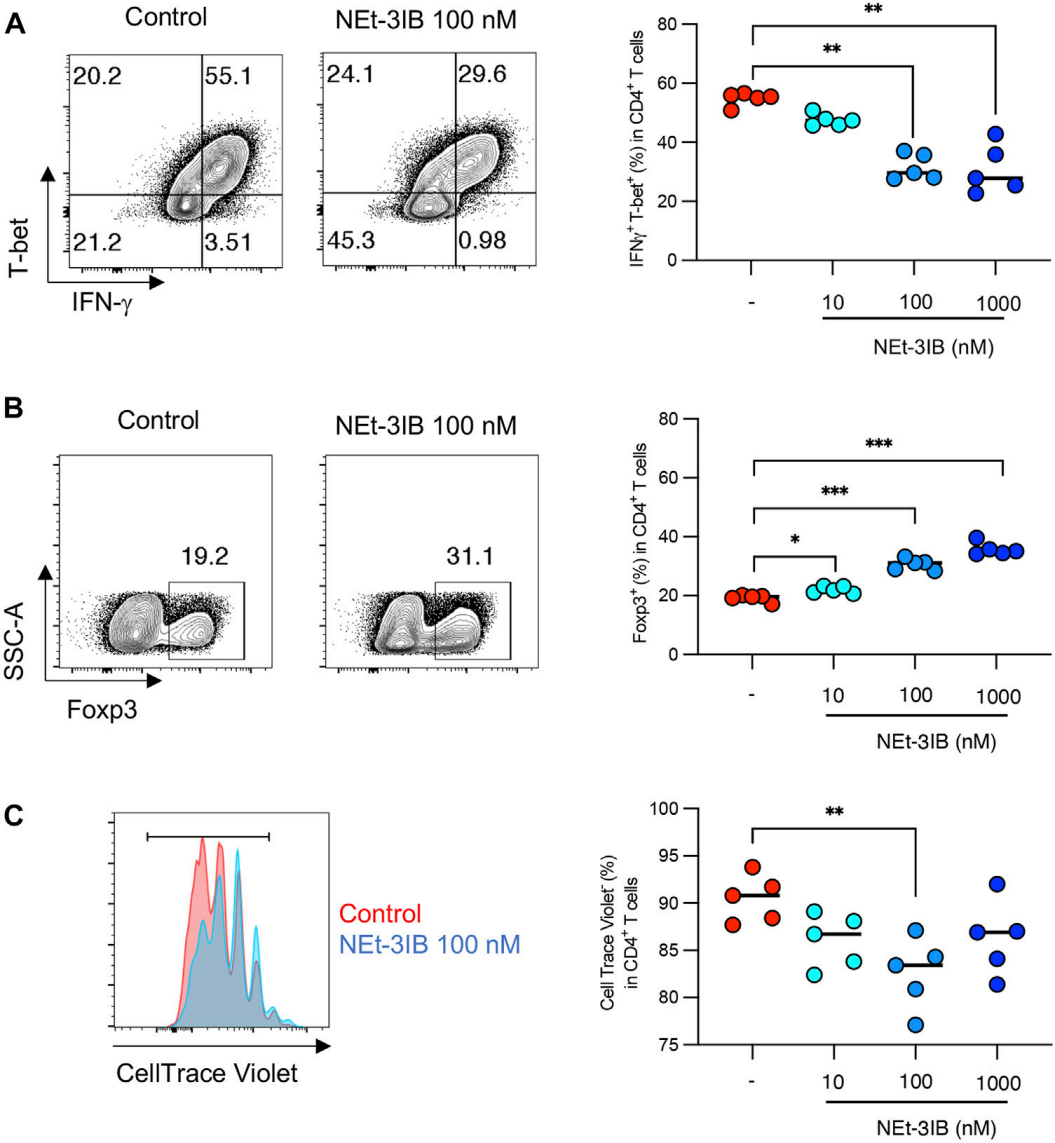
NEt-3IB inhibits differentiation of inflammatory T cells and induces Foxp3^+^ cells in Th1 and Th17 conditions. **(A, B)** Naïve T cells were cultured in Th1 conditions, with or without NEt-3IB treatment for 5 days and analyzed for T-bet and IFN-γ expression **(A)**, and for Foxp3 expression in the CD4^+^ T cell population **(B)**. **(C)** Naïve T cells were labeled with CellTrace Violet and cultured in Th1 conditions with or without NEt-3IB treatment for 5 days. Cultured cells were analyzed for their degree of proliferation in the CD4^+^ T-bet^+^ IFN-γ^+^ population. *p < 0.05, **p < 0.01, ***p < 0.005.

We further investigated whether NEt-3IB has a direct impact on the differentiation of Th cells using an *in vitro* culture system. Sorted naïve CD4^+^ T cells were cultured under Th1-skewed conditions for 5 days with or without NEt-3IB treatment before flow cytometric analysis for the expression of Th1 markers (T-bet, IFN-γ), and a Treg marker (Foxp3). We found that the frequencies of the T-bet^+^ and IFN-γ^+^ cells decreased in the NEt-3IB-treated group in a dose-dependent manner (Supplementary Figure S2D and Figure 4A). Surprisingly, NEt-3IB treatment increased the proportion of Foxp3^+^ T cells in a dose-dependent manner despite culture in Th1 conditions (Supplementary Figure S2E and Figure 4B).

We subsequently examined whether NEt-3IB affected the proliferation of Th1 cells *in vitro*. Fluorescent dye-labeled naïve CD4^+^ T cells were cultured under Th1-differentiating conditions for 5 days with or without Net-3IB treatment, and then the dilution of CellTrace Violet was analyzed to detect proliferating cells. NEt-3IB treatment significantly reduced the frequency of proliferating cells in the T-bet^+^IFN-γ^+^ Th1 population when compared with the vehicle control (Supplementary Figure S2F and Figure 4C). Together, these data suggest that NEt-3IB directly inhibits both the differentiation and proliferation of Th1 cells.

## Discussion

In this study, we demonstrated that NEt-3IB attenuated the development of wasting disease and colitis in T cell-dependent experimental colitis models by inhibiting the accumulation of Th1 cells, which is the most dominant cell type contributing to the pathogenesis of CD. Furthermore, our *in vitro* data revealed that NEt-3IB directly inhibited the differentiation and expansion of Th1 cells. NEt-3IB was designed to have lower lipophilicity compared with conventional RXR agonists such as bexarotene (Takamatsu et al., 2008; Kobayashi et al., 2015). We confirmed that NEt-3IB exhibits a lower plasma concentration compared with bexarotene after oral administration. In the detailed pharmacokinetics analysis after i.v. administration, the distribution to the liver represented approximately 49% of the total dosage of NEt-3IB, while in the kidney there was only 12% of the total dosage. Considering that the concentration of NEt-3IB in bile rises after its peak in the liver, NEt-3IB is mostly likely to be excreted efficiently to the bile. Notably, we observed that 60% of the total dosage reached the colon within 24 h post-oral administration, after the bile concentration peaked at 4 h. These data support the enterohepatic circulation of NEt-3IB, consistent with our previous report (Kobayashi et al., 2015). In contrast, such enterohepatic circulation was not observed in bexarotene, which was readily absorbed in the upper intestine after oral administration with only a small portion delivered to the colon. Thus, NEt-3IB is efficiently delivered to the colonic lumen and should be translocated to the cLP under inflammatory conditions, which are often associated with mucosal barrier disruption.

Early studies showed that the activation and overexpression of certain nuclear receptors exert anti-inflammatory effects. For instance, PPAR agonists suppress the differentiation of Th1 cells *in vitro* by inhibiting the JAK–STAT signaling pathway (Natarajan and Bright, 2002). LXR agonists also display similar effects on the differentiation of Th1 cells (Solt et al., 2012). Moreover, PPARγ and LXR agonists both suppress the proliferation of T cells (Yang et al., 2000; Cunard et al., 2002; Diab et al., 2004; Bensinger et al., 2008; Solt et al., 2012). Given that these nuclear receptors heterodimerize with RXR, and RXR agonists can activate these partner nuclear receptors, the inhibitory effect of NEt-3IB on Th1 response may depend on the activation of LXR and PPAR signaling. Additionally, PPARγ agonists inhibit IL-12 production by macrophages, which is important for the differentiation and survival of Th1 cells (Natarajan and Bright, 2002). Thus, NEt-3IB may directly and indirectly suppress the Th1 response through the inhibition of IL-12 production by macrophages.

Our *in vitro* experiments demonstrated that Foxp3^+^ cells arose in the NEt-3IB-treated group despite the Th1-differentiation conditions. Given that RXR agonists modulate Foxp3^+^ Treg cell differentiation (Takeuchi et al., 2013), NEt-3IB may drive naïve CD4^+^ T cells to upregulate Foxp3 regardless of the culture conditions. Because ectopic Foxp3 expression represses IFN-γ expression, it is possible that NEt-3IB reduced the number of Th1 cells *in vitro* by downregulating T-bet expression. Nevertheless, in the CD4^+^CD45RB^high^ T-cell-transfer colitis model, no significant difference in the frequency of Treg cells was identified between the control and NEt-3B-treated groups. This observation implies that NEt-3IB may repress IFN-γ expression via an unknown mechanism other than upregulation of Foxp3. For instance, NEt-3IB may indirectly dampen Th1 cell expansion by diminishing Th17 cell differentiation. We observed a reduction in IFN-γ-producing Th17 cells in the cLP of the NEt-3IB-treated group. This transient cell population is generated from conventional Th17 cells and further differentiates into Th1 cells (Sujino et al., 2011; Basdeo et al., 2017). Therefore, the reduction in IFN-γ-producing Th17 cells in the cLP of the NEt-3IB group cells may lead to a decrease in Th1 cells. Notably, RXR and LXR agonists negatively regulate the differentiation of Th17 cells *in vitro* (Solt et al., 2012; Takeuchi et al., 2013). Thus, NEt-3IB possibly restricted the development of Th17 cells in the colitis model mice, resulting in the reduction of IFN-γ-producing Th17, and in turn, Th17 cell-derived Th1 cells.

NEt-3IB treatment also reduced the number of macrophages. NEt-3IB is likely to indirectly suppress the infiltration of macrophages by inhibiting the expansion of IFN-γ-producing Th17 and Th1 cells. Furthermore, NEt-3IB may directly diminish macrophage activation. In support of this, we previously found that a partial RXR agonist, CBT-PMN, downregulates the production of pro-inflammatory cytokines by bone marrow-derived macrophages (Onuki et al., 2018). Furthermore, other groups showed that FXR agonist treatment significantly downregulated IFN-γ-related gene expression (Renga et al., 2009), and that PPAR and LXR agonists also suppress the production of inflammatory cytokines from macrophages (Birrell et al., 2007; Paukkeri et al., 2013) Additionally, overexpression of NR4A1 and NR4A2 in human macrophages inhibits pro-inflammatory cytokines (Bonta et al., 2006). All these nuclear receptors form heterodimers with RXR and are activated by RXR agonists. These facts support the possibility that NEt-3IB directly inhibits the activation and cytokine production of macrophages by activating RXR partner nuclear receptors.

In conclusion, the present study demonstrated that NEt-3IB ameliorated colitis by inhibiting both the expansion of Th1 cells and the activation of inflammatory macrophages locally in the colon. Our results indicate that NEt-3IB is a promising candidate for IBD treatment as a small molecule inhibitor.

## Data Availability

The original contributions presented in the study are included in the article Supplementary Material, further inquiries can be directed to the corresponding authors.
