# Supplementary material for: A Retinoid X Receptor Agonist Directed to the Large Intestine Ameliorates T-Cell-Mediated Colitis in Mice
Source: Front Pharmacol. 2021 Aug 12;12:715752. doi: 10.3389/fphar.2021.715752 (PMC8406631; doi:10.3389/fphar.2021.715752)
Supplement: Supplementary file 1 [file DataSheet1.PDF]

Supplementary Fig. 1

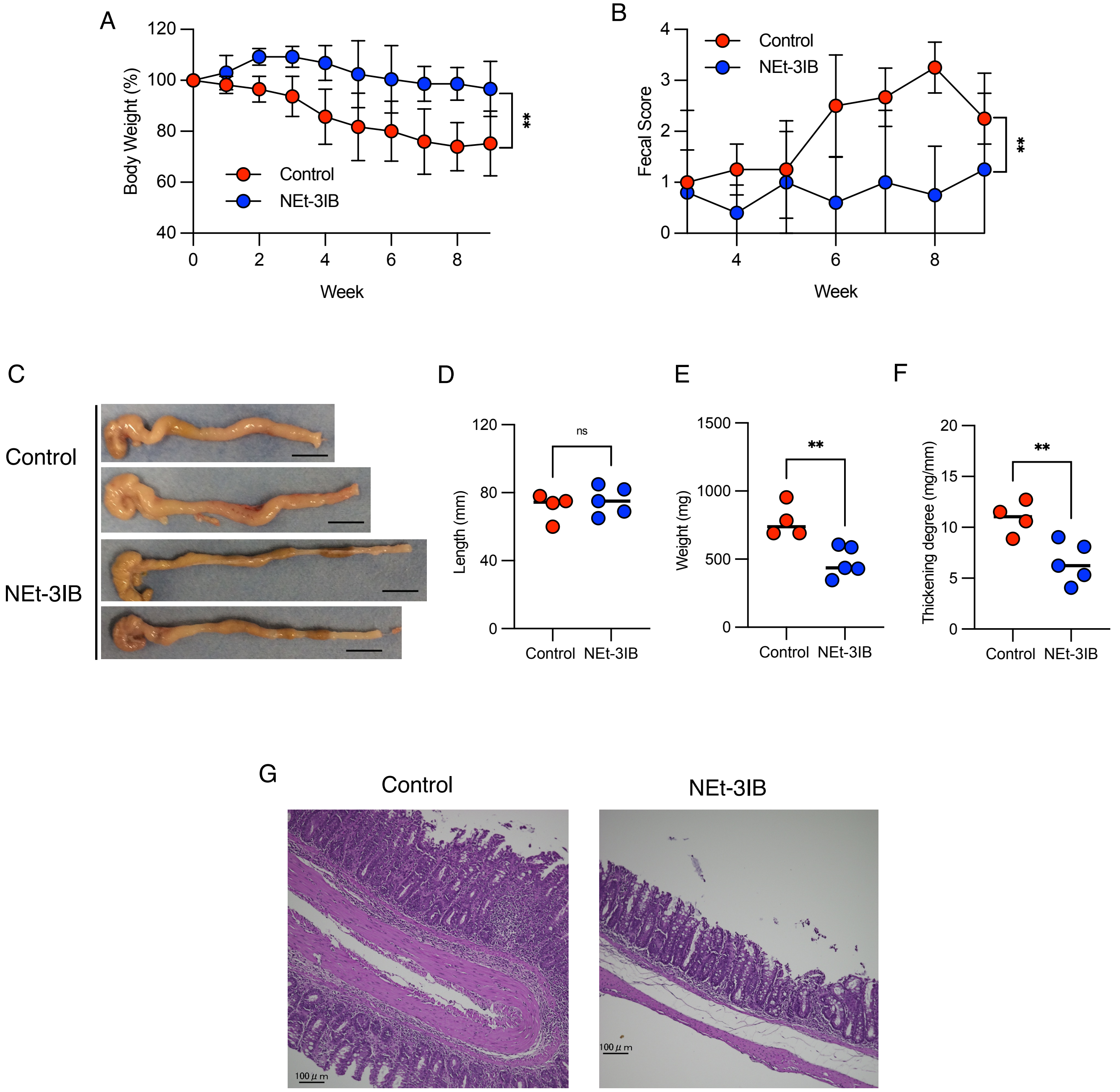

NEt-3IB ameliorates T-cell-dependent experimental colitis in *Rag1$^{-/-}$* mice

A-G, Experimental colitis was induced by adoptive transfer of CD4^{+} CD45RB^{high} T cells in *Rag1$^{-/-}$* mice fed with control or NEt-3IB containing diet for 9 weeks and observed body weight loss **(A)** and fecal diarrhea score **(B)**. Data shown are the average. Area under the curve of both body weight change and fecal score, and then the statistical analysis was performed. **(C-F)** Colon length, colon weight and colon thickening were observed and measured in week 9 . Scale bar : 1 cm. **(G)** Colonic specimen were stained with haematoxylin and eosin (HE) . * $P < 0.05$, ** $P < 0.01$

Supplementary Fig. 2

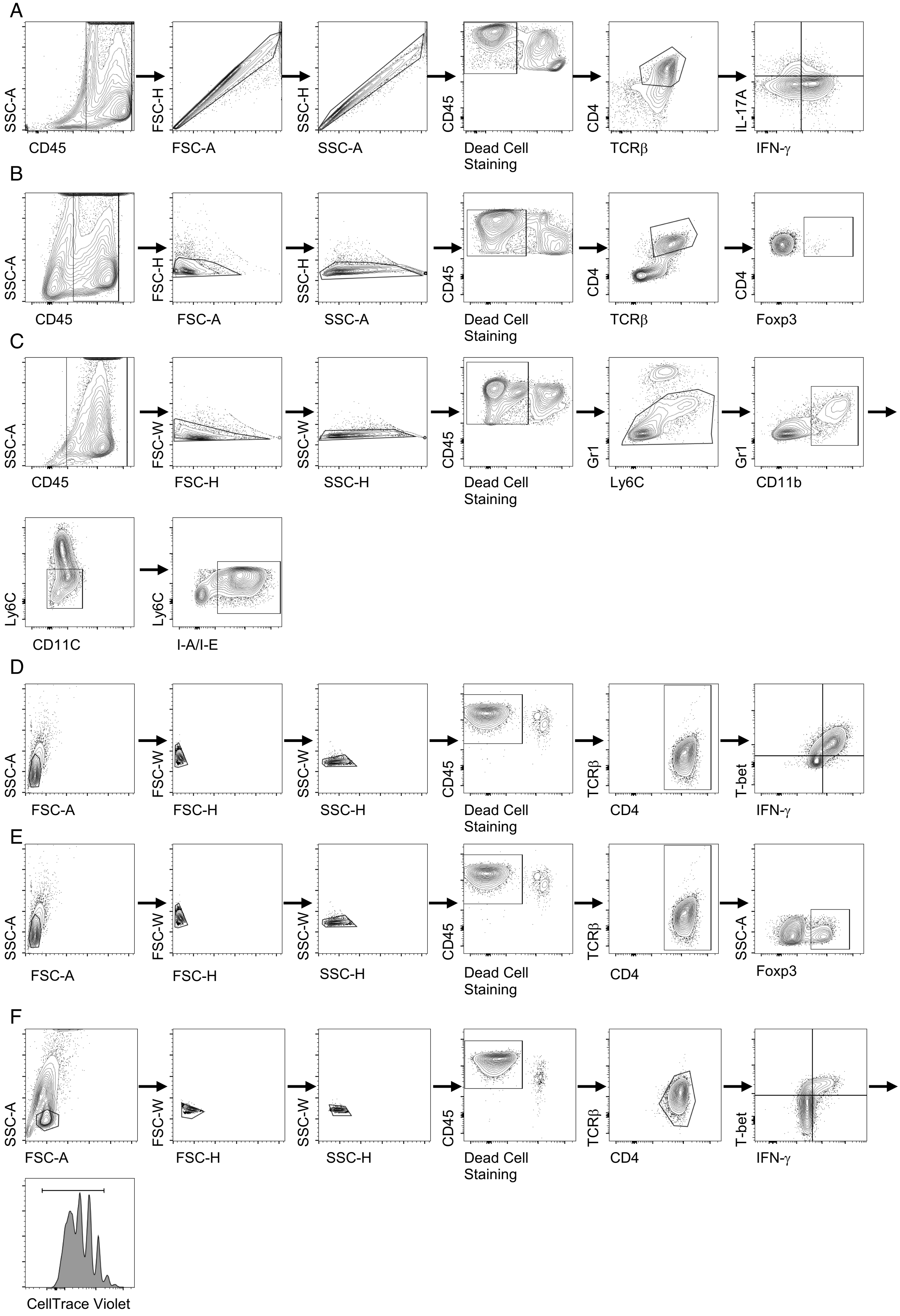

Gating strategy used for flow cytometry analysis.
(A) Gating strategy for Th1, Th17 and IFN-g-producing Th17 cells of colitis model mice presented on Figure 3A and B. **(B)** Gating strategy for Treg cells of colitis model mice presented on Figure 3C. **(C)** Gating strategy for CD11b^{+} Ly6G^{-} Ly6C^{-} CD11C^{-} MHC2^{+} macrophages of colitis model mice presented on Figure 3D. **(D)** Gating strategy for cultured Th1 cells presented on Figure 4A. **(E)** Gating strategy for Foxp3 expressing T cells cultured under Th1 polarizing condition cells presented on Figure 4B. **(F)** Gating strategy to analyze proliferation rate of cultured Th1 cells with CellTrace Violet presented on Figure 4C.
